# Nitric Oxide Protects L-Type Calcium Channel of Cardiomyocyte during Long-Term Isoproterenol Stimulation in Tail-Suspended Rats

**DOI:** 10.1155/2015/780814

**Published:** 2015-06-22

**Authors:** Zhi-Jie Yue, Peng-Tao Xu, Bo Jiao, Hui Chang, Zhen Song, Man-Jiang Xie, Zhi-Bin Yu

**Affiliations:** Department of Aerospace Physiology, Fourth Military Medical University, No. 169 Changlexi Road, Xi'an 710032, China

## Abstract

The aim of this study was to investigate the effects of nitric oxide (NO) and reactive oxygen species (ROS) on L-type calcium channel (LTCC) gating properties of cardiomyocytes during long-term isoproterenol (ISO) stimulation. Expression and activity of nNOS as well as* S*-nitrosylation of LTCC *α*1C subunit significantly decreased in the myocardium of SUS rats. Long-term ISO stimulation increased ROS in cardiomyocytes of SUS rats. ISO-enhanced calcium current (*I*_Ca,L_) in the SUS group was less than that in the CON group. The maximal *I*_Ca,L_ decreased to about 80% or 60% of initial value at the 50th minute of ISO treatment in CON or SUS group, respectively. Specific inhibitor NAAN of nNOS reduced maximal *I*_Ca,L_ to 50% of initial value in the CON group; in contrast, NO donor SNAP maintained maximal *I*_Ca,L_ in SUS group to similar extent of CON group after 50 min of ISO treatment. Long-term ISO stimulation also changed steady-state activation (*P* < 0.01), inactivation (*P* < 0.01), and recovery (*P* < 0.05) characteristics of LTCC in SUS group. In conclusion, NO-induced* S*-nitrosylation of LTCC *α*1C subunit may competitively prevent oxidation from ROS at the same sites. Furthermore, LTCC can be protected by NO during long-term ISO stimulation.

## 1. Introduction

Exposure to microgravity can lead maladaptive alterations in multiple organ systems of human body. When returned to Earth, the astronauts could not stand motionless for 10 min without experiencing symptoms of dizziness or presyncope, which is defined as orthostatic intolerance [1]. Altered baroreflex reactivity [2], cardiac pump function [3, 4], volume-regulating systems [5], and vascular function and reactivity through nitric oxide synthase-dependent mechanisms [6] are thought to be contributors to orthostatic intolerance. Among these contributors, a marked decrease in cardiac output and adrenergic responsiveness of cardiovascular system is the main reason [1, 4, 7]. In order to maintain blood pressure stability during stand stress, sympathetic activity and blood catecholamine levels compensatively increased [1]. Although weightlessness or simulated weightlessness enhances *β*-adrenergic receptor responsiveness in the heart [8], the myocardial contractility still decreases during isoproterenol (ISO) stimulation [9]. The ISO-enhanced L-type Ca^2+^ current (*I*_Ca,L_) in cardiomyocytes is significantly decreased in 4-week tail-suspended rats [7, 10]. ISO binds the *β*-adrenergic receptor to activate protein kinase A (PKA) which mediates phosphorylation of L-type calcium channel (LTCC). PKA-dependent phosphorylation of LTCCs causes a several-fold increase in *I*_Ca,L_ [11]. While the ISO-PKA signaling pathway has not changed in cardiomyocytes of tail-suspended rats [9], the reduction of intracellular peak calcium transients indicates an alteration in LTCCs characteristics under ISO stimulation. Overstimulation and/or long-term stimulation of *β*-adrenergic receptor can injure LTCCs [12]. However, nitric oxide (NO) plays an important role in protective LTCCs during *β*-adrenergic receptor stimulation, but the underlying mechanism is still unclear [13].

Except inducible NO synthase (iNOS), both endothelial (eNOS) and neuronal (nNOS) NO synthases are constitutively expressed in distinct subcellular locations within cardiomyocytes under a physiological condition [14]. In cardiomyocytes, eNOS primarily localized to caveola indirectly inhibits *I*_Ca,L_ through cGMP-protein kinase G downstream signaling pathway and reducing responsiveness of cardiomyocytes to ISO [15]. The nNOS localized to sarcolemma and sarcoplasmic reticulum directly inhibits *I*_Ca,L_ through protein thiol nitrosylation (*S*-nitrosylation) downstream signaling pathway and increasing responsiveness of LTCC to ISO [16]. Therefore, nNOS-derived NO may be a major regulator to *I*_Ca,L_ and responsiveness of LTCC to ISO in cardiomyocytes. On the other hand, angiotensin II and long-term ISO stimulation increase reactive oxygen species (ROS) in cardiomyocytes [17, 18]. High dose of ROS can induce oxidation of cysteine residues on LTCC to decrease responsiveness of *I*_Ca,L_ to ISO in cardiomyocytes [19]. The oxidation and* S*-nitrosylation may be competitively exerted at the same sites of cysteine residues on LTCC. So we need to elucidate the interaction between NO and ROS in regulating *I*_Ca,L_ and responsiveness of LTCC to ISO and, furthermore, to elucidate the mechanism in which NO protects the gating properties of LTCC during long-term or intensive ISO stimulation in cardiomyocytes in tail-suspended rats.

## 2. Methods

### 2.1. Animal Model

Adult male Sprague-Dawley rats weighing 220~250 g were maintained on a 12 : 12 h light-dark cycle and fed standard pellet diet. The room temperature was 22°C ± 2°C and relative humidity was about 50%. The experimental procedures were approved by the Fourth Military Medical University Animal Care and Use Committee. The rats were randomly divided into 6 groups: 1-week (1 wk SUS), 2-week (2 wk SUS), and 4-week (4 wk SUS) tail-suspended groups and their synchronous control groups (1 wk CON, 2 wk CON, and 4 wk CON). The tail-suspended rats were kept in 30° head-down tilt and hindlimb unloading position [4]. Soleus muscle was weighed to confirm the efficacy of model at the end of experiment.

### 2.2. Isolation of Left Ventricular Cardiomyocytes

Cardiomyocytes were enzymatically isolated from rat heart [20]. After heparinization (300 IU/100 g body weight) for 30 min, rats were anesthetized with an intraperitoneal injection of pentobarbital sodium (40 mg/kg). The heart was quickly excised and cannulated via the aorta in a Langendorff apparatus. Modified Joklik's MEM (Sigma-Aldrich, St. Louis, MO, USA) containing 10 mM HEPES sodium and 0.1% bovine serum albumin (BSA, Sigma-Aldrich) at pH 7.30 (solution A) was first used to wash remaining blood in coronary vessels for 5 min. Then the heart was digested for about 30 min with perfusion of solution B (1 mg/mL collagenase I (Sigma-Aldrich) in solution A). At the end of digestion, the heart was perfused with solution A again for 5 min to wash out collagenase I and to terminate digestion. The heart was rapidly minced; then high-quality isolated cardiomyocytes were collected and preserved in solution C (solution A containing 1% BSA). The concentration of Ca^2+^ in solution C containing cardiomyocytes was gradually restored to 1.25 mM. And then calcium-tolerant cardiomyocytes were obtained and preserved for the following experiments.

### 2.3. Reactive Oxygen Species Staining

Superoxide anion radical production in the cardiomyocytes was detected by dihydroethidium (DHE) staining (Sigma-Aldrich). Cardiomyocytes were treated with or without 1 *μ*M ISO for 30 minutes before loading the myocytes with DHE (2 *μ*M) at 37°C for 30 minutes. Fluorescent images were obtained with an Olympus FV1000 confocal microscope (Tokyo, Japan). The number of positively stained nuclei of cardiomyocytes was counted from 5 randomly selected fields per heart.

### 2.4. Western Blotting and Detection of* S*-Nitrosylation

The heart was rapidly excised after euthanasia and perfused with an oxygenated (95% O_2_-5% CO_2_) Krebs-Henseleit solution [4] or with 1 *μ*M isoproterenol (ISO; Sigma-Aldrich) or with 100 *μ*M S-nitroso-N-acetyl penicillamine (SNAP, a NO donor; Sigma-Aldrich) in Langendorff mode for 60 minutes, respectively. The protein expression was measured by western blotting analysis as described previously [4]. Thirty mg of myocardium was taken from left ventricle and homogenized with 600 *μ*L of lysis buffer containing (in mM) 50 Tris-HCl, 0.1% Triton X-100, 1 EDTA, 50 NaF, 0.2 PMSF, 10 *β*-glycerophosphate, 0.2 Na_3_VO_4_, and 5 sodium pyrophosphate at pH 7.40 by a homogenizer (POLYTRON PT-MR 2100, Kinematica, Switzerland). The muscle protein extracts were resolved by SDS-PAGE using Laemmli gel. 8% gel with an acrylamide/bisacrylamide ratio of 37.5 : 1 was used for the examination of LTCC *α*1C subunit and 10% gel for the examination of nNOS, eNOS, and iNOS. After electrophoresis, proteins in the gel were electrically transferred to nitrocellulose membrane (0.45 *μ*m pore size) with a Bio-Rad semidry transfer apparatus. The blotted nitrocellulose membranes were blocked with 1% BSA in Tris-buffered saline (150 mM NaCl, 50 mM Tris-HCl, and pH 7.5) and cut into two parts. The upper membranes were incubated with a rabbit polyclonal anti-eNOS (1 : 500; Santa Cruz Biotechnology (SCB), Inc., CA, USA), mouse monoclonal anti-nNOS antibody (1 : 2500; Transduction Laboratories, Lexington, Kentucky, USA), rabbit polyclonal anti-iNOS antibody (1 : 500; SCB), rabbit polyclonal anti-*α*1C antibody (1 : 1000; SCB), and rabbit polyclonal anti-phospho-Ser1928 antibody (1 : 500; Badrilla Ltd., Leeds, UK) in Tris-buffered saline containing 0.1% BSA at 4°C overnight. The lower membranes were incubated with mouse monoclonal anti-*β*-actin (1 : 4000; Sigma-Aldrich). The nitrocellulose membranes were incubated with IRDye 680CW goat-anti-mouse or IRDye 800CW goat-anti-rabbit secondary antibodies (1 : 10 000) for 90 min at room temperature and visualized using an Odyssey scanner (LI-COR Biosciences, Lincoln, NE, USA).

Protein* S*-nitrosocysteine posttranslational modification was detected by a modified* S*-nitrosylation switch assay (*S*-Nitrosylation Western Blot Kit, Pierce, Rockford, IL, USA) as described previously [21]. Quantitative analysis of western blots was performed with the NIH Image J software.

### 2.5. Detection of NOS Activity

The activity of NOS was determined by a NOS Assay Kit (Sigma-Aldrich) using cardiac muscle protein extracts as described previously [22]. As depicted above, the protein concentration of myocardial extract was determined by a Bradford method and then was adjusted to 1 *μ*g/*μ*L in each sample. The 10 *μ*L muscle extract was added into a 96-well flat-bottomed plate; then 90 *μ*L testing buffer and 100 *μ*L testing reaction solutions were added into each well. Blank control well was added with 10 *μ*L homogenization buffer, 90 *μ*L testing buffer, and 100 *μ*L testing reaction solution. All samples were incubated at 37°C for 2 h. After incubation, the fluorescence intensity was detected through microplate system (FL800, BioTek, Winooski, VT, USA) at 485 nm excitation wavelength and 512 nm emission wavelength. The fluorescence intensity was recorded as the relative fluorescence unit 1 (RFU1) in the sample well and as RFU2 in blank control well. The relative NOS activity was determined by the following formula: RFU = RFU1 − RFU2.

Myocardial extracts were assayed under a variety of conditions: with or without nNOS inhibitor {(4S)-N-(4-amino-5[aminoethyl]aminopentyl)-N′-nitroguanidine, NAAN, 240 nM; Calbiochem, Darmstadt, Germany} in the absence of CaCl_2_ and with or without L-arginine. Two hundred and forty nM of NAAN can completely inhibit the RFU.

### 2.6. Electrophysiological Measurements

Cardiomyocytes were placed in a chamber on a heated stage (30°C) of inverted microscope and perfused with extracellular solution. As described previously [23], the *I*_Ca,L_ was recorded using the conventional whole-cell voltage-clamp technique with a MultiClamp 700B patch-clamp amplifier (Axon instruments, Union City, CA, USA). Patch electrode was fabricated from borosilicate glass with a Micropipette Puller Model 97 (Sutter Instrument, Novato, CA, USA), and its resistance was 1.5~2.5 MΩ when the electrode was filled with pipette solution. The membrane was sucked to form the whole-cell configuration with a giga seal. Cell capacitance was calculated by integrating current elicited by 5 mV depolarization from a holding potential of −80 mV. The experiment was conducted with series resistance compensated by 80% and capacitance compensation. Protocol and data acquisition was performed with pCLAMP software (Version 10.0, Axon Instruments, USA). Currents were filtered at 1.02 kHz and digitized at 10 kHz. At the end of experiments, data were analyzed offline with pCLAMP 10.0.

The pipette solution contained (in mM) 150 CsCl, 1 MgCl_2_, 10 EGTA, 5 HEPES, 5 Na_2_ATP, and 5 Na_2_-creatine phosphate, equilibrated with 95% O_2_-5% CO_2_, pH 7.2 by titration with CsOH [23]. The extracellular solution contained (in mM) 133.5 NaCl, 4 CsCl, 1.8 CaCl_2_, 1.2 MgCl_2_, 10.0 HEPES, and 11.1 glucose (pH was adjusted to 7.4 with NaOH). Thirty *μ*M tetrodotoxin (TTX) was added in the extracellular solution to block the Na^+^ current. After recording the basic *I*_Ca,L_, 1 *μ*M ISO, 240 nM nNOS inhibitor NAAN, or 100 *μ*M SNAP was added in the chamber. The *I*_Ca,L_ was recorded at the 10th and 50th minute, respectively, after applying different compounds.

Cardiomyocyte was depolarized from a holding potential of −80 mV to −40 mV for 200 ms and then depolarized periodically to vary the testing potentials between −50 mV and 60 mV for 400 ms in an increment of 10 mV. Every pulse was elicited at 0.1 Hz to ensure recovery of LTCC. The steady-state activation and inactivation curves were obtained with individual two-pulse protocols (Figures 5(a) and 6(a)) and fitted with the Boltzmann equations [24]. The time course of recovery from inactivation of *I*_Ca,L_ was determined by another double-pulse protocol, which stepped from a holding potential of −80 mV to −40 mV for 200 ms, followed by a step to 0 mV for 400 ms, maintained at −40 mV (the first pulse, *I*_Ca,L1_). At different interval the second pulse (*I*_Ca,L2_) stepped to 0 mV for 400 ms and returned to −80 mV. There is 10 s between every double-pulse to ensure complete recovery of *I*_Ca,L_ (Figure 7(a)). Recovery ratio of *I*_Ca,L_ can be calculated with the following equation [25]:

(1) $$\begin{array}{c} \text{Recovery ratio }I\left(\;\%\;\right)=\frac{I_{\text{Ca,L2}}}{I_{\text{Ca,L1}}}, \end{array}$$

where *I*_Ca,L1_ is the *I*_Ca,L_ evoked by the first pulse and *I*_Ca,L2_ is the *I*_Ca,L_ evoked by the second pulse.

The curve of recovery can be plotted as recovery ratio at different interval, which is fitted by a single-exponent equation:

(2) $$\begin{array}{c} I_{i}\left(\;\%\;\right)=A+B\exp\left(\;-\frac{t_{i}}{\tau }\;\right), \end{array}$$

where *τ* is the time constant, *A* is the offset value, *B* is amplitude of time-dependent component, and *t*_*i*_ is the interval between the first pulse and the second pulse.

### 2.7. Statistical Analysis

Data are presented as mean ± SEM. Differences between every two groups were compared by the paired Student's *t*-test. For multigroup comparisons, two-way ANOVA followed by Tukey post hoc test was performed. A value of *P* < 0.05 was considered statistically significant.

## 3. Results

### 3.1. Expression and Activity of nNOS in Left Ventricular Myocardium of Tail-Suspended Rats

**Figure 1:**
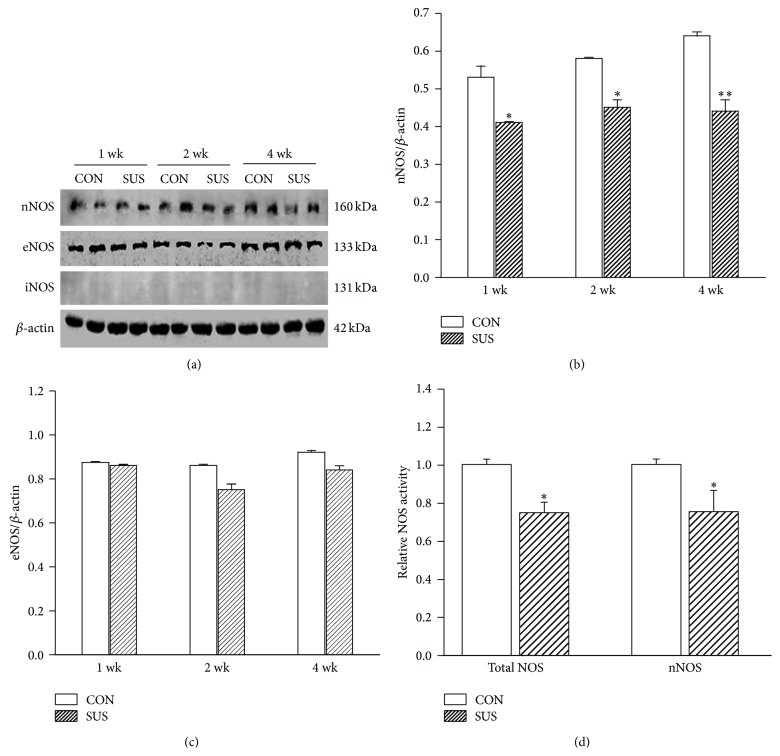
NOSs expression of myocardium in 1-, 2-, and 4-week tail-suspended rats and NOS activity in myocardium of 4-week tail-suspended rats. (a) Representative western blots of eNOS, nNOS, and iNOS. *β*-actin is an internal control. (b) Ratios of nNOS to *β*-actin in tail-suspended (SUS) and the synchronous control (CON) rats. (c) Ratios of eNOS to *β*-actin. (d) Total NOS and nNOS activity in the myocardium of 4-week tail-suspended and control rats. Data are mean ± SEM; *n* = 6 hearts. ^*∗*^ *P* < 0.05, ^*∗∗*^ *P* < 0.01* versus* synchronous CON.

The ratio of soleus muscle wet weight to body weight showed a significant decrease in 4 wk SUS group (0.19 ± 0.01) as compared with the synchronous control value (0.40 ± 0.01; *P* < 0.01). It indicated the efficacy of tail suspension. Western blots showed that nNOS and eNOS, but no iNOS, were constitutively expressed in left ventricular myocardium of both control (CON) and tail-suspended (SUS) groups (Figure 1(a)). There was a significant decrease in expression of nNOS in 1 wk, 2 wk, and 4 wk SUS groups (*P* < 0.05 or *P* < 0.01, Figure 1(b)). Expressions of eNOS in the myocardium were unaltered in 1 wk, 2 wk, and 4 wk SUS groups compared with the CON groups (Figure 1(c)). The activity of total NOS and specific activity of nNOS only reduced in the 4 wk SUS group compared with the CON group (*P* < 0.05, Figure 1(d)). Therefore, we focused on 4 wk SUS group to observe the changes in calcium channel properties of cardiomyocytes.

### 3.2. Expression, Phosphorylation, and* S*-Nitrosylation of LTCC *α*1C Subunit

**Figure 2:**
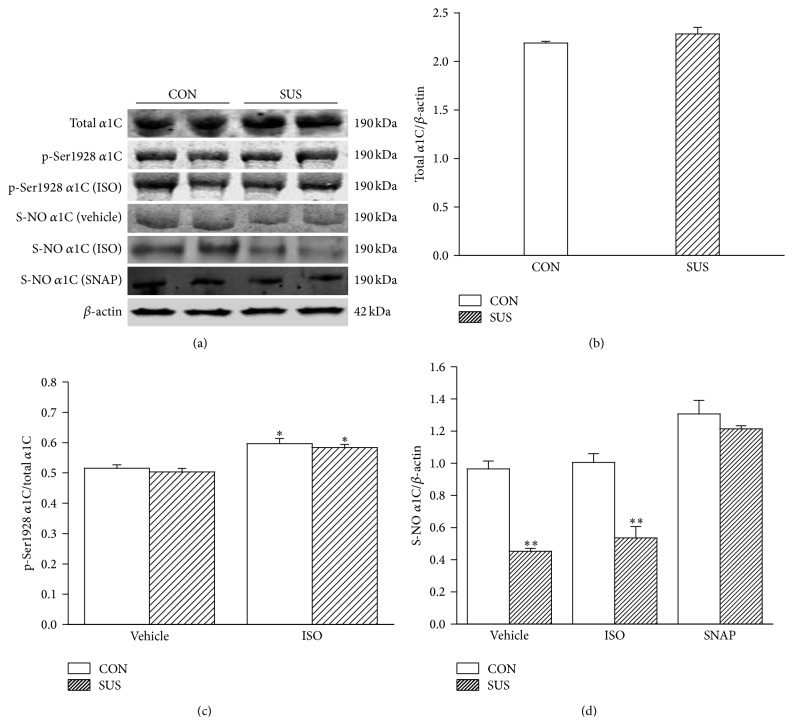
Expression, phosphorylation at Ser1928, and* S*-nitrosylation of L-type calcium channel *α*1C subunit. (a) Representative western blots of LTCC *α*1C subunit in 4-week tail-suspended (SUS) and synchronous control (CON) rats. (b) Ratios of LTCC *α*1C subunit to *β*-actin. (c) Ratios of p-Ser1928 to total *α*1C subunit. (d) Ratios of* S*-nitrosylation of LTCC *α*1C to *β*-actin. Data are mean ± SEM; *n* = 3 hearts in each group. ^*∗*^ *P* < 0.05 or ^*∗∗*^ *P* < 0.01* versus* synchronous CON.

The expression of LTCC *α*1C subunit was not changed in left ventricular myocardium of 4 wk SUS group compared with the CON group (Figures 2(a) and 2(b)). ISO increased the phosphorylation level at Ser1928 of LTCC *α*1C subunit in both CON and SUS groups (*P* < 0.05, Figure 2(c)). But the level of phosphorylation at Ser1928 between the CON and SUS group was not altered before or after ISO treatment.* S*-nitrosylation of LTCC *α*1C subunit showed a decrease in left ventricular myocardium of the SUS group compared with the CON group with or without ISO treatment (*P* < 0.01, Figure 2(d)), but SNAP completely restored* S*-nitrosylation of LTCC *α*1C subunit in 4 wk SUS group (*P* < 0.01, Figure 2(d)).

### 3.3. Superoxide Anion Radical Production in the Cardiomyocytes

**Figure 3:**
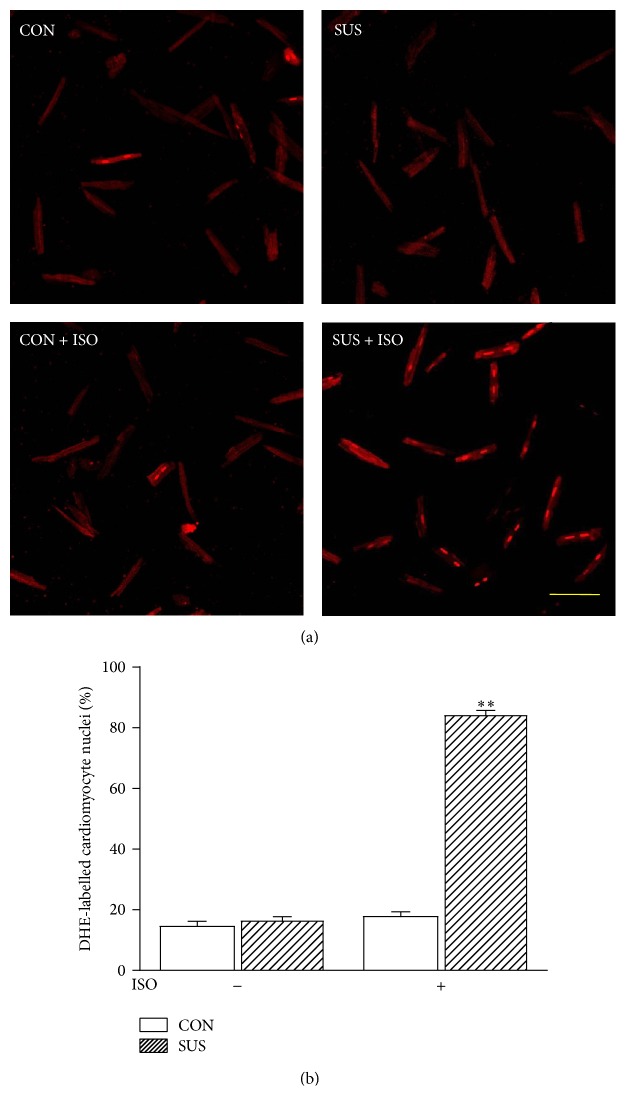
Superoxide anion radical production in left ventricular cardiomyocyte of CON and SUS rats. (a) Confocal fluorescence microscopy images of isolated cardiomyocytes from CON and SUS rats probed with DHE dye for assessment of superoxide anion radical production. Myocytes were treated with ISO (1 *μ*M) at 37°C for 30 min. Scale bar: 100 *μ*m. (b) Quantitation of the DHE-staining nuclei in cardiomyocytes from different groups. Data are mean ± SEM; *n* equals at least 150 cardiomyocytes from 3 hearts. ^*∗∗*^ *P* < 0.01* versus* synchronous CON.

The superoxide anion radical production was detected by DHE staining in cardiomyocytes treated with ISO. As shown in Figure 3, there was no difference in superoxide anion radical production between CON and SUS cardiomyocytes under the basic condition. After ISO treatment, the superoxide anion radical production in SUS cardiomyocytes was significantly enhanced as compared with that in the CON cardiomyocytes (*P* < 0.01, Figures 3(a) and 3(b)).

### 3.4. Responsiveness of LTCC Current (*I*_Ca,L_) to ISO under Different Conditions

**Figure 4:**
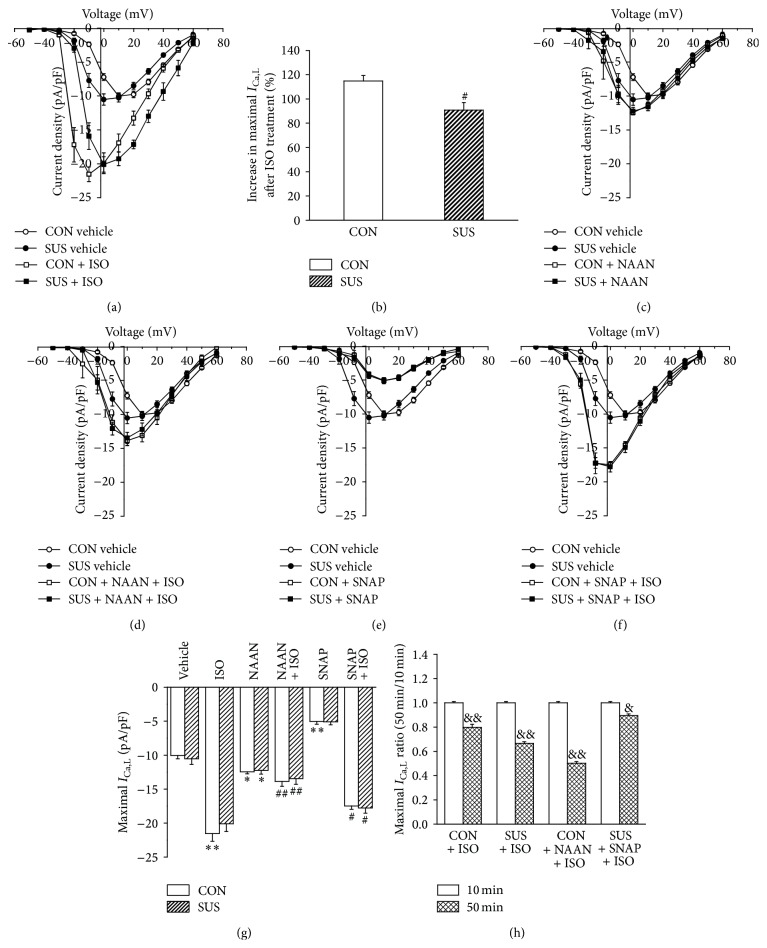
*I*-*V* curves in left ventricular cardiomyocyte of CON and SUS rats. (a)* I-V* curves of the CON and 4 wk SUS groups with or without 1 *μ*M of ISO stimulation. (b) The increasing percentage of peak *I*_Ca,L_ with 1 *μ*M of ISO treatment for 10 min. (c)* I-V* curves of LTCC with or without 240 nM nNOS inhibitor (NAAN). (d)* I-V* curves of LTCC with or without 240 nM NAAN + 1 *μ*M ISO treatment. (e)* I-V* curves of LTCC with or without 100 *μ*M NO donor (SNAP) treatment. (f)* I-V* curves of LTCC with or without 100 *μ*M SNAP + 1 *μ*M ISO treatment. (g) Maximal *I*_Ca,L_ of* I-V* curves with different treatments. (h) Ratios of peak *I*_Ca,L_ at the 50th min with ISO treatment to that at the 10th min. Data are mean ± SEM; *n* = 6 cardiomyocytes from 5 hearts in each treatment. ^*∗*^ *P* < 0.05 and ^*∗∗*^ *P* < 0.01* versus* CON vehicle. ^#^ *P* < 0.05 and ^##^ *P* < 0.01* versus* CON with ISO treatment. ^&^ *P* < 0.05 and ^&&^ *P* < 0.01* versus *the value at the 10th min.

The voltage dependence of *I*_Ca,L_ activation showed a left shift in the SUS group as compared with the CON group under the basic condition. ISO increased maximal *I*_Ca,L_ in the CON and SUS groups and only induced a left shift in the CON group (Figures 4(a) and 4(g)). The percent increase in the maximal amplitude of *I*_Ca,L_ in the CON group was greater than that in the 4 wk SUS group after ISO stimulation (*P* < 0.05, Figure 4(b)). Under the NAAN treatment, the maximal *I*_Ca,L_ significantly increased in the CON and SUS group (*P* < 0.05, Figures 4(c) and 4(g)); ISO induced less enhancement in the maximal *I*_Ca,L_ and did not shift the* I-V* curves in the CON and SUS groups (*P* < 0.01, Figures 4(d) and 4(g)). SNAP decreased maximal *I*_Ca,L_ in the CON and SUS groups but did not influence* I-V* curves (*P* < 0.01, Figures 4(e) and 4(g)). ISO also induced less enhancement in the maximal *I*_Ca,L_ during SNAP treatment in the CON and SUS groups (*P* < 0.05, Figures 4(f) and 4(g)).

The maximal *I*_Ca,L_ at the 10th minute of ISO treatment was used as 100% in all of the groups. The maximal *I*_Ca,L_ at the 50th minute of ISO treatment reduced to 80% or 60% in the CON or SUS group, respectively. The maximal *I*_Ca,L_ at the 50th minute of ISO treatment further reduced to 50% in the CON with NAAN treatment group (*P* < 0.01). SNAP resisted the reduction in the maximal *I*_Ca,L_ at the 50th minute of ISO treatment in the SUS group (*P* < 0.05, Figure 4(h)).

### 3.5. Normalized Steady-State Activation Curves of LTCC

**Figure 5:**
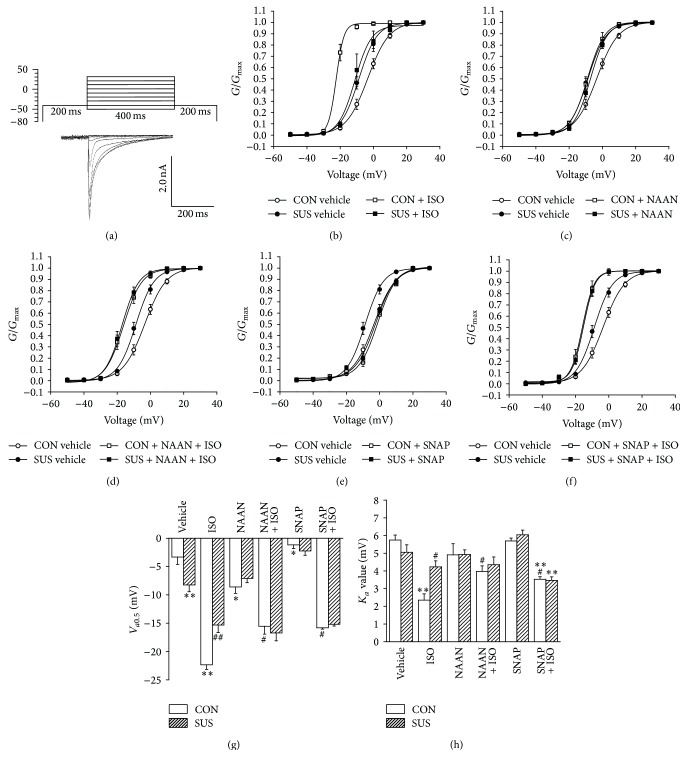
Properties of LTCC steady-state activation in cardiomyocytes of 4-week tail-suspended and control rats. (a) Special protocol was designed to analyze LTCC steady-state activation. (b) Steady-state activation curves of LTCC with or without 1 *μ*M ISO treatment in CON and 4 wk SUS groups. *G* is the conductance of LTCC at different testing potential; *G*_max⁡_ is the maximum conductance. (c) Steady-state activation curves of LTCC with or without 240 nM NAAN treatments. (d) Steady-state activation curves of LTCC with 240 nM NAAN + 1 *μ*M ISO treatments. (e) Steady-state activation curves of LTCC with 100 *μ*M SNAP. (f) Steady-state activation curves of LTCC with 100 *μ*M SNAP + 1 *μ*M ISO. (g) *V*_*a*0.5_ of steady-state activation curves with different treatments. *V*_*a*0.5_ is the half-activated potential which reflects the open probability of channels. (h) *K*_*a*_ value of steady-state activation curves. *K*_*a*_ is a slope factor which indicates the voltage sensitivity of channels. Data are mean ± SEM; *n* = 6 cardiomyocytes from 5 hearts. ^*∗*^ *P* < 0.05 and ^*∗∗*^ *P* < 0.01* versus* CON vehicle. ^#^ *P* < 0.05 and ^##^ *P* < 0.01* versus* CON with ISO treatment.

The steady-state activation curve of LTCC had a leftward shift in 4 wk SUS group compared with CON group. ISO caused more leftward shift of LTCC activation curve in the CON group, but not in the SUS group (Figure 5(b)). The open probability of LTCC (*V*_*a*0.5_) increased (*P* < 0.01) and the voltage sensitivity of LTCC (*K*_*a*_ value) unaltered in the SUS group compared with the CON group (Figures 5(g) and 5(h)). ISO induced greater open probability of LTCC in the CON group than that in the SUS group (*P* < 0.01, Figure 5(g)). ISO also caused a higher voltage sensitivity of LTCC in the CON group than that in the SUS group (*P* < 0.05 or *P* < 0.01, Figure 5(h)). NAAN increased the open probability of LTCC in the CON group (*P* < 0.05, Figures 5(c) and 5(g)) but unchanged the voltage sensitivity of LTCC in both groups (Figures 5(c) and 5(h)). On the contrary, SNAP decreased the open probability of LTCC (*P* < 0.05) and unchanged the voltage sensitivity of LTCC in both groups (Figures 5(e), 5(g), and 5(h)). ISO induced less increase in LTCC open probability during NAAN or SNAP treatments than under basic condition in the CON group (Figures 5(d), 5(f), and 5(g)). The voltage sensitivity of LTCC was not influenced in the combination treatment of NAAN and ISO (Figures 5(d) and 5(h)) but increased in the combination treatment of SNAP and ISO (*P* < 0.01, Figures 5(f) and 5(h)).

### 3.6. Normalized Steady-State Inactivation Curves of LTCC

**Figure 6:**
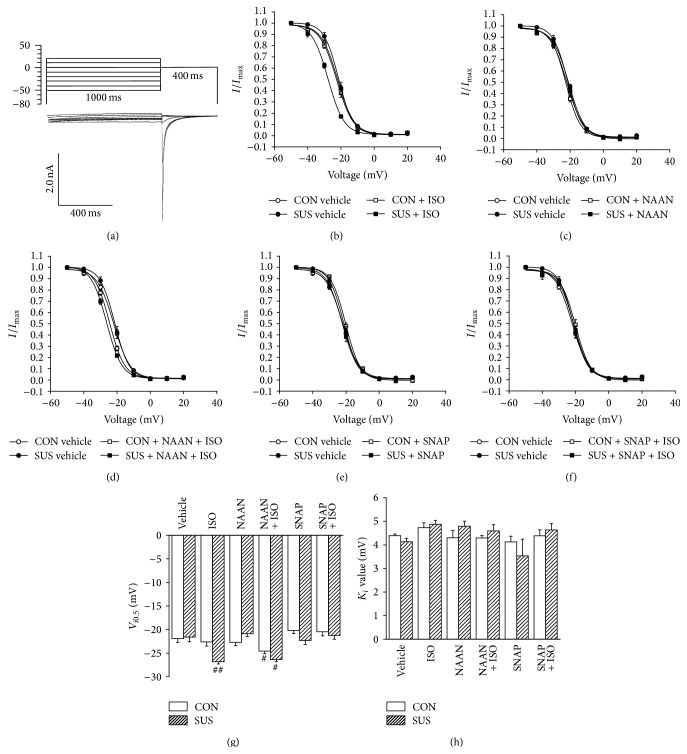
Properties of LTCC steady-state inactivation in cardiomyocytes of 4-week tail-suspended and control rats. (a) Special protocol was designed to analyze LTCC inactivation property. (b) Steady-state inactivation curves of LTCC with or without 1 *μ*M ISO treatment. *I* is *I*_Ca,L_ at different prepulse potential; *I*_max⁡_ is the maximum current. (c) Steady-state inactivation curves of LTCC with or without 240 nM NAAN. (d) Steady-state inactivation curves of LTCC with or without 240 nM NAAN + 1 *μ*M ISO. (e) Steady-state inactivation curves of LTCC with or without 100 *μ*M SNAP. (f) Steady-state inactivation curves of LTCC with or without 100 *μ*M SNAP + 1 *μ*M ISO. (g) *V*_*i*0.5_ of steady-state inactivation curves with different treatments in CON and 4 wk SUS groups. *V*_*i*0.5_ is the prepulse potential at which 50% of channels are available. (h) *K*_*i*_ value of steady-state inactivation curves. *K*_*i*_ is a slope factor which indicates voltage sensitivity of the channels. Data are mean ± SEM; *n* = 6 cardiomyocytes from 5 hearts. ^#^ *P* < 0.05 and ^##^ *P* < 0.01* versus* CON with ISO treatment.

Steady-state inactivation curve of LTCC had no shift between the CON and SUS groups. ISO induced a leftward shift of LTCC inactivation curve in the SUS group (Figure 6(b)). There were no influences of NAAN (Figure 6(c)), SNAP (Figure 6(e)), NAAN + ISO (Figure 6(d)), and SNAP + ISO (Figure 6(f)) treatments on *K*_*i*_ values in the CON and SUS groups (Figure 6(h)). The *V*_*i*0.5_ of LTCC inactivation curve decreased to more negative potential in the SUS group with ISO treatment (*P* < 0.01) or in the CON and SUS groups with NAAN plus ISO treatment (*P* < 0.05, Figure 6(g)).

### 3.7. Recovery Curves of LTCC from Inactivation

**Figure 7:**
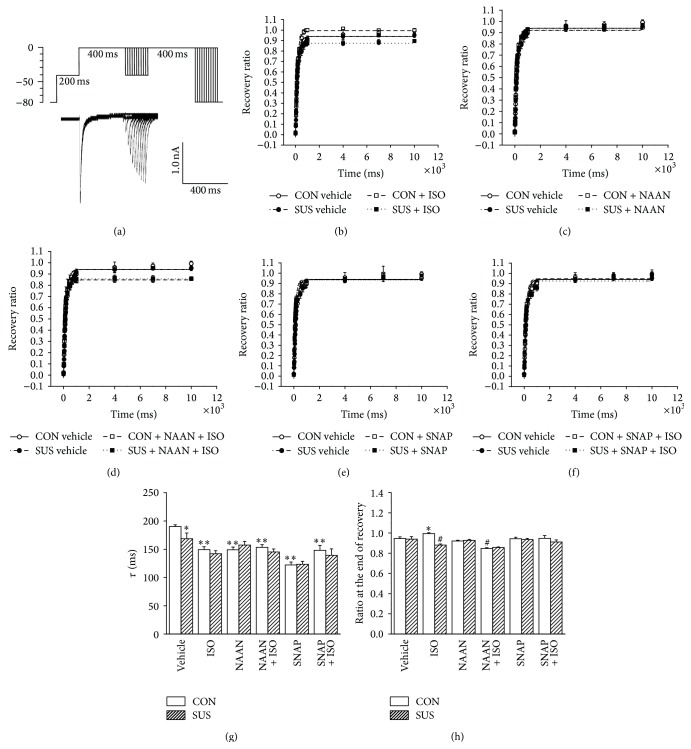
Properties of LTCC recovery in cardiomyocytes of 4-week tail-suspended and control rats. (a) Protocol for LTCC recovery and representative evoked currents. (b) Recovery curves of LTCC with or without 1 *μ*M ISO treatment. (c) Recovery curves of LTCC with or without 240 nM NAAN. (d) Recovery curves of LTCC with or without 240 nM NAAN + 1 *μ*M ISO treatment. (e) Recovery curves of LTCC with or without 100 *μ*M SNAP treatment. (f) Recovery curves of LTCC with or without 100 *μ*M SNAP + 1 *μ*M ISO treatment. (g) *τ* values of recovery curves with different treatments in CON and 4 wk SUS groups. *τ* is a time constant which indicates the recovery rate of inactivated LTCC. (h) Ratios at the end of recovery. Data are mean ± SEM; *n* = 6 cardiomyocytes from 5 hearts. ^*∗*^ *P* < 0.05 and ^*∗∗*^ *P* < 0.01* versus* CON vehicle. ^#^ *P* < 0.05* versus* CON with ISO treatment.

The recovery rate of inactivated LTCC, indicated by *τ* time constant, accelerated but the percentage of recovery at the eleventh second unaltered in the SUS group compared with the CON group (Figures 7(b), 7(g), and 7(h)). ISO accelerated the recovery rate of inactivated LTCC in both CON and SUS groups (Figures 7(b) and 7(g)). ISO increased the percentage of recovery at the eleventh second in the CON group, but not in the SUS group (Figures 7(b) and 7(h)). Under the nNOS inhibition condition, the percentage of recovery at the eleventh second was decreased during ISO stimulation in the CON and SUS group (Figures 7(c), 7(d), and 7(h)). While NO was increased by SNAP, ISO accelerated the recovery rate of inactivated LTCC but unaltered the percentage of recovery at the eleventh second in the CON and SUS group (Figures 7(e), 7(f), 7(g), and 7(h)).

## 4. Discussion

In the present study the results showed for the first time that expression and activity of nNOS were decreased in the myocardium of 4 wk tail-suspended rats. The reduced nNOS-derived NO decreased* S*-nitrosylation of LTCC *α*1C subunit in the SUS group. In contrast, ISO induced more superoxide anion radical production in cardiomyocytes of SUS rats. Because* S*-nitrosylation of LTCC *α*1C subunit can prevent oxidation of LTCC from reactive oxygen species and protects the gating property of LTCC during long-term ISO stimulation, LTCC gating property of cardiomyocytes under the basic condition and during ISO treatment was damaged in the SUS group.

*β*-adrenergic stimulation leads to 7-fold increase in calcium current of cardiomyocyte LTCC, mediated by protein kinase A (PKA) phosphorylation. The cleaved *α*1C subunit lacking its C-terminal tail is not a substrate for phosphorylation by PKA [26], whereas the full-length *α*1C subunit was readily phosphorylated on serine 1928 (Ser1928) in the C-terminal domain [27]. PKA phosphorylation of *α*1C at Ser1700 does not have a major role in *β*-adrenergic stimulation of Ca^2+^ current in the adult murine heart [28]. Therefore, LTCC *α*1C Ser1928 is a main site of PKA phosphorylation in cardiomyocytes. Yang and colleagues identified Ser1928 as the residue that is phosphorylated by PKC* in vitro* and* in vivo* [29]. Protein kinase G (PKG) also phosphorylated the Ser1928 on the *α*1C subunit [30]. Therefore, the Ser1928 on the *α*1c subunit is a common site for LTCC phosphorylation by PKA, PKC, and PKG. In the present study we observed that ISO increased the Ser1928 phosphorylation of LTCC *α*1C subunit in the CON and SUS groups, but there was no difference in the level of Ser1928 phosphorylation between two groups in the basic condition and ISO stimulation (Figure 2). Based on the important effect of the LTCC *α*1C subunit Ser1928 phosphorylation on *I*_Ca,L_, the reduced responsiveness of *I*_Ca,L_ to ISO in cardiomyocytes of the SUS rats might be involved in other mechanisms.

Except that the phosphorylation of protein modulates *I*_Ca,L_ and responsiveness of LTCC to ISO in cardiomyocytes, oxidation and nitrosylation of protein are also important regulators of LTCC. Cysteine residues in proteins are the most likely target of redox or nitrosylation modification. There are 48 cysteine residues on the *α*1C subunit of LTCC in the rat. Not all of these, especially location on transmembrane I, II, III, and IV domains, will be susceptible to redox or nitrosylation. The C-terminal tail concluding 17 cysteine residues has high probability for redox or nitrosylation despite the fact that the exact sites are not revealed [31]. Because cysteine residues on LTCC C-terminal tail are adjacent to phosphorylated sites, the redox or nitrosylation of cysteine residues regulates not only gating properties of LTCC but also responsiveness of LTCC to *β*-adrenergic receptor stimulation. However, the mechanisms underlying interaction between redox and nitrosylation to regulate LTCC are not resolved.

The function of the LTCC can be modified during changes in cellular redox state. While exogenous or endogenous ROS exceeds the threshold in cardiomyocytes, ROS may induce the oxidation of cysteine residues on C-terminal tail of LTCC and further changes gating properties of LTCC. The *I*_Ca,L_ increases, but responsiveness of LTCC to ISO decreases in cardiomyocytes after transient H_2_O_2_ treatment [19]. Long-term ISO stimulation markedly increased superoxide anion radical productions in cardiomyocytes of 4 wk SUS rats (Figure 3). The oxidative stress might be involved in regulating LTCC gating properties in cardiomyocytes of SUS rats. In contrast, NO directly inhibits LTCC via protein thiol nitrosylation [32]. In fact, nitrosylation is a process to scavenge ROS in cardiomyocytes [33]. On the other hand, the nitrosylated cysteine residues of LTCC are unavailable for oxidation by ROS. Therefore, nitrosylation of *α*1C subunit protects the gating properties of LTCC. Because high cytoplasmic concentration of myoglobin can scavenge NO, the effective distance of NO within cardiomyocytes is likely to be limited to a local environment. Since nNOS localizes closely to LTCC in cardiomyocytes [34], the nNOS-derived NO is a major factor on LTCC gating properties [35]. In the present study,* S*-nitrosylation of LTCC decreased due to a reduced expression and activity of nNOS in the SUS group (Figures 1 and 2). The responsiveness of *I*_Ca,L_ to ISO was decreased in the SUS group. In particular, *I*_Ca,L_ decreased markedly during long-term ISO stimulation compared with the control group (Figure 4). NO donor SNAP could reverse the nitrosylation of LTCC in the SUS group to the control level; responsiveness of *I*_Ca,L_ to ISO was increased and *I*_Ca,L_ could maintain the similar extent during long-term ISO stimulation (Figures 1 and 4). While NOS was inhibited by NAAN in the control myocardium, the changes were similar to those of the SUS group: the decreased responsiveness of LTCC to ISO and reduced *I*_Ca,L_ after long-term ISO stimulation (Figure 4). The above results indicate that the decreased nNOS activity combined with the elevated ROS in cardiomyocytes of SUS rats reduces the responsiveness of LTCC to ISO through modulating the gating properties of LTCC.

Due to the C-terminal tail of LTCC having a high probability to be nitrosylated or oxidized, NO and ROS can modulate the gating properties of LTCC. There are three states of LTCC: activation, inactivation, and deactivation (recovery) [36]. Sequential transition among states maintains the gating properties of LTCC and changes of three states will have an impact on *I*_Ca,L_ [36]. Compared with the control group, LTCC in cardiomyocytes of SUS rats showed a slow activation, accelerated inactivation, and incomplete recovery during ISO stimulation. Inhibition of NOSs in the control group showed similar changes in gating properties of LTCC with the SUS group. In contrast, SNAP reserved the gating properties of LTCC in cardiomyocytes of SUS rats to the control level. During long-term ISO stimulation, elevated ROS induced more oxidation on less nitrosylated LTCC in cardiomyocytes of SUS rats. It led to high open probability of LTCC but accelerated inactivation and insufficient recovery of LTCC. Finally, *I*_Ca,L_ was markedly decreased during long-term ISO stimulation. Therefore, ISO did not exert sufficient compensation to orthostatic intolerance.

In conclusion, NO can scavenge in part ROS in cardiomyocytes and NO-induced* S*-nitrosylation of LTCC *α*1C subunit prevents LTCC from oxidation by reactive oxygen species. Therefore, NO protects the gating property of LTCC in cardiomyocytes during long-term ISO stimulation.
